# Genomic evolution and ecotype divergence in thraustochytrids: insights from comparative genomics and phylogenomics

**DOI:** 10.3389/fmicb.2025.1608951

**Published:** 2025-06-30

**Authors:** Yingying Wen, Xingyu Zhu, Jiaqian Li, Xiuping Liu, Qixuan Li, Guangyi Wang

**Affiliations:** ^1^Center for Marine Environmental Ecology, School of Environmental Science and Engineering, Tianjin University, Tianjin, China; ^2^Key Laboratory of Systems Bioengineering (Ministry of Education), Tianjin University, Tianjin, China

**Keywords:** thraustochytrids, whole genome sequencing, comparative genomics, ecotype differentiation, fatty acid biosynthesis

## Abstract

**Background:**

Thraustochytrids are unicellular heterotrophic protists within the Stramenopiles group, widely distributed across marine ecosystems. Understanding the mechanisms underlying their metabolic ecotype evolution is pivotal for revealing how these organisms drive the marine carbon cycle and adapt to diverse environments.

**Methods:**

In this study, we report a high-quality genome of *Aurantiochytrium* sp. TWZ-97 and conduct a comparative genomics analysis of thraustochytrid strains to investigate ecotype-specific differences in genome structure, evolutionary-developmental relationships, and core functional genes.

**Results:**

Comparative genomics revealed that “anabolic” strains (TWZ-97, Mn4, SW8) possess larger genomes with lower gene density, whereas “catabolic” strains (S-28, S-429) have smaller, gene-rich genomes with stable repetitive elements. Phylogenetic analyses revealed that the “anabolic” strains diverged relatively recently, around 2.389 million years ago, while the “catabolic” strains evolved independently for over 190.7 million years, reflecting prolonged, lineage-specific adaptation. Functionally, “anabolic” strains were enriched in fatty acid synthase genes, whereas hydrolytic enzyme genes were unique to the “catabolic” strains. Both ecotypes exhibited a significant abundance of fatty acid desaturase (FAD) genes, and polyketide synthase (PKS) genes displayed unique long sequences, multi-domain architectures, and ecotype-specific gene differentiation patterns.

**Conclusion:**

Together, this study provides crucial molecular evidence for the genetic basis of metabolic specialization and ecotype diversification in thraustochytrids.

## 1 Introduction

Thraustochytrids, a group of unicellular heterotrophic protists widely distributed in marine ecosystems, not only play a vital role in organic matter decomposition, potentially fulfilling multiple ecological functions and serve as an important microbial resource for the industrial production of polyunsaturated fatty acids (PUFAs) and additionally, they may fulfill multiple ecological functions (Morabito et al., 2019; Liu et al., 2023). Recent studies suggest that thraustochytrids may have evolved two ecotypes: “anabolic” and “catabolic” strains (Song et al., 2018; Liu et al., 2023). As a key nutrient source for high trophic level organisms, the “anabolic” strains are characterized by a well-developed lipid synthesis pathway, efficient accumulation of polyunsaturated fatty acids and other high-value metabolites (Song et al., 2018). In contrast, the “catabolic” strains are characterized by the specific expression of hydrolytic enzymes such as cellulase and xylanase, facilitating the degradation of particulate organic matter (Liu et al., 2023); and driving the conversion of organic carbon into dissolved forms, thereby acting as and driving the conversion of organic carbon into dissolved forms, thereby acting as a “carbon cycle engine.” However, the genomic mechanisms underlying the differentiation of these ecotypes—including genome evolution strategies, phylogenetic relationships, and metabolic regulation—remain poorly understood, limiting predictions of their ecological roles and biotechnological potential.

Genomic studies of thraustochytrids have primarily focused on the biosynthesis of PUFA products such as docosahexaenoic acid (DHA) and eicosapentaenoic acid (EPA). The genome size of thraustochytrids typically ranges from 30 to 60 Mb (Ji et al., 2015; Liu et al., 2016; Zhao et al., 2016; Hu et al., 2020; Liang et al., 2020), and there are significant differences in the genome size of different ecotypes of thraustochytrids, with the genome size of “anabolic” strains (~60 Mb) being almost twice as large as that of “catabolic” strains (~30 Mb) (Liu et al., 2023). These larger genomes may be due to more genomic repetitive sequences or unassembled sequences (Iwasaka et al., 2018). It is well established that microbial genome characteristics closely relate to their ecological niches: where adaptive evolution often coincides with increased genome plasticity (e.g., transposon mutagenesis or gene family amplification), while stable ecological niches may favor genome streamlining (Cicconardi et al., 2023; Matti et al., 2023; Xiao et al., 2025). Advances in genome sequencing technologies have enabled detailed investigation of the functional genes in thraustochytrids, particularly, those involved in PUFA biosynthesis. While interspecies variation in lipid-accumulation capacity has been observed, the genetic basis for these differences remains unanswered (Li-Beisson et al., 2019; Morabito et al., 2019). In particular, differences in fatty acid synthesis in the fatty acid synthase (FAS) and polyketide synthase (PKS) pathways between ecotypes at the genomic level have yet to be fully elucidated.

The phylogenetic relationships of thraustochytrids have also been contentious, with numerous thraustochytrid taxa exhibiting ambiguous polyphyly (Dellero et al., 2018a). Traditional classification based on their combined morphological features, PUFA content and carotenoids is not sufficient to accurately delineate species boundaries. This is because the same morphological features can be found in different genera. For example, the genus *Ulkenia* is characterized by an amoeboid stage, but amoeboid cells can also be found in species belonging to the genus *Aurantiochytrium* (Dellero et al., 2018b) and *Thraustochytrium* (Bongiorni et al., 2005). In addition, carotenoid and PUFA profiles are also influenced by different growth conditions (temperature, medium composition) in terms of synthesizing and accumulating (Bowles et al., 1999). With the development of sequencing technology, the integration of genomic, morphological, and physiological data offers a more robust approach for resolving phylogenetic relationships in this group of marine protists (Yokoyama and Honda, 2007). Notably, the biosynthetic and degradative capabilities of thraustochytrids are directly related to their taxonomy, with ecotype-specific metabolic functions emerging over evolutionary time.

In this study, we report the results of a high-quality genome assembly of high-yielding fatty acid thraustochytrids: *Aurantiochytrium* sp. TWZ-97. We performed comparative genomic analyses, phylogenetic reconstruction, and functional gene module analyses across “anabolic” (TWZ-97, Mn4 and SW8) and “catabolic” (S-28, S-429) strains. Our obejectives are to: (1) elucidate the adaptive significance of the genome evolutionary patterns of the two types of strains, (2) investigate the correlation between their divergence time and ecotype formation, and (3) explore the functional differentiation and synergistic mechanism governing fatty acid synthesis. The study further evaluates the ecological functions and biotechnological potentials of these ecotypes, offering new insights into the evolution of metabolic networks in marine eukaryotic microorganisms.

## 2 Materials and methods

### 2.1 Strains

The strain used in this study, *Aurantiochytrium* sp. TWZ-97, was isolated from the mangrove area of Hainan, China (Zhang et al., 2019), and was selected for whole genome sequencing. The strain was identified by PCR amplification and sequence analysis of its full-length 18S rRNA gene. Isolated strains were kept on modified Vishniac’s (MV) agar medium (glucose, 10 g/L; yeast extract, 0.1 g/L; peptones, 1.5 g/L; agar, 20 g/L; and artificial sea salt, 33 g/L) at 28°C.

### 2.2 Genome sequencing and assembly

The strain TWZ-97 was cultivated in culture medium (M4) (glucose, 20 g/L; yeast extract, 1 g/L; peptones, 1.5 g/L; KH_2_PO_4_, 0.25 g/L; and artificial sea salt, 33 g/L) with reciprocal shaking (170 rpm) at 28°C for 4 days. Cells were then harvested from 200 mL of fresh culture and genomic DNA was extracted using the cetyltrimethylammonium bromide (CTAB) method (Aboul-Maaty and Oraby, 2019). The amount and quality of DNA was determined by NanoDrop One spectrophotometer (NanoDrop Technologies, Wilmington, DE), Qubit 3.0 fluorometer (Life Technologies, Carlsbad, CA, USA), and agarose gel electrophoresis in a commercial company (Lianchuan Biotechnology Co., Ltd., Hangzhou, China).

Whole genome sequencing of TWZ-97 was performed on NovaSeq 6,000 (Illumina, USA) and PromethION (Oxford Nanopore Technologies, Oxford, UK) sequencing platforms (Myers et al., 2000). The software Fastp and Oxford Nanopore GUPPY were used for quality assessment and data filtering. Q-value is an important indicator of sequencing quality. The higher the quality value, the lower the likelihood of incorrect sequencing. The second and third generations filter out low-quality sequences based on Q-value less than or equal to 5 and less than or equal to 7, respectively. GenomeScope (version 1.0) (Vurture et al., 2017) and Jellyfish (version 2.2.10) (Marçais and Kingsford, 2011) were used to estimate genome size and heterozygosity. NECAT (Chen et al., 2021) was used to perform hybrid Illumina + Nanopore assembly. Racon (version 1.4.11) (Vaser et al., 2017) was used to perform error correction on three-generation sequencing data; Pilon (version 1.23) (Walker et al., 2017) was used to perform error correction on second-generation sequencing data on the preliminary assembly results after three-generation error. Finally, heterozygosity was removed to obtain the final assembly data. Genomic integrity was assessed by BUSCO (version 4.1.2) (Simão et al., 2015) using a eukaryotic model. Genome sequencing and assembly were performed at Lianchuan Biotechnology Co., Ltd. (Hangzhou, China).

### 2.3 Gene prediction and functional annotation

In this study, gene structure prediction was performed using a combination of homology prediction, ab initio prediction, and transcript prediction. Among them, homology prediction was done using Exonerate, ab initio prediction was done using Augustus (version 3.3.2), Genscan (version 1.0), and GlimmerHMM (version 3.0.4), and RNA-seq data was reconstructed through stringtie (version 2.1.4) to obtain transcripts, and then coding frames were predicted using TransDecoder (version 5.1.0) (Song et al., 2018; Liu et al., 2023). MAKER (version 2.31.10) (Carson and Mark, 2011) was used to integrate gene sets predicted by various methods. The protein sequences encoded by the genes in the gene set were annotated to the genes using the available protein databases Uniprot, Non-Redundant Protein Sequence Database (NR), Kyoto Encyclopedia of Genes and Genomes (KEGG) and Gene Ontology (GO) databases. Where KEGG annotation was performed using KOBAS association to KEGG ORTHOLOGY as well as PATHWAY. The Uniprot database records the correspondence of each protein family to a functional node in Gene Ontology, by which the biological function performed by a gene-encoded protein sequence was predicted by this system. Gene prediction and annotation were performed at Lianchuan Biotechnology Co., Ltd. (Hangzhou, China).

### 2.4 Comparative genome analysis

To understand the genomic information in thraustochytrids, comparative genomic analyses of *Botryochytrium* sp. S-28 (S-28), *Oblongichytrium* sp. S-429 (S-429), *Aurantiochytrium* sp. Mn4 (Mn4), *Aurantiochytrium* sp. SW8 (SW8) and TWZ-97 were performed via the OrthoVenn3 server^1^ (Sun et al., 2023). Phylogenetic analyses and gene family contraction and expansion analyses were performed using the OrthoVenn3 built-in species database. TBtools (version 2.154) (Chen et al., 2023) was used for collinearity analysis of TWZ-97, Mn4, SW8, S-28 and S-429.

### 2.5 Identification of gene families and their bioinformatics analysis

The hidden Markov model (HMM) files corresponding to the structural domains of FA_desaturase (PF 00487), FA_desaturase 2 (PF 03405), and TMEM189 (PF 10520) of FAD, as well as those corresponding to PKS (cd 00833), downloaded from Pfam protein family database^2^ were searched against the protein data of TWZ-97, S-28 and S-429 with e-value ≤1e^−5^ as criterion (Zhiguo et al., 2019; Ahmadizadeh et al., 2020; Hajiahmadi et al., 2020; Cheng et al., 2022; Waheed et al., 2024). Then, the SMART database^3^ was used to confirm each putative gene. ExPASy (), DeepTMHMM 1.0,^4^ SignalP 5.0^5^ and CELLO v.2.5^6^ were used to analyze the physicochemical properties, transmembrane structural domains, signaling peptides and subcellular localization of FAD and PKS members, respectively. Conserved motifs were identified using the online analysis tool MEME^7^ with parameters set to standard settings. Raw GFF files containing genome annotation data were manipulated to obtain gene structures. Conserved structural domains were identified by the online tool NCBI-CDD (Conserved Domain Database).^8^ All results of these analyses were generated using TBtools (Chen et al., 2023). The amino acid sequences of FAD and PKS gene family proteins were analyzed by multiple sequence comparison using the built-in ClustalW program of MEGA 11 (Koichiro et al., 2021), and the results were used to construct a phylogenetic tree by the neighbor-joining (NJ) method and to perform a bootstrap evaluation (Bootstrap), which was repeated 1,000 times, and the missing values were handled by the pairwise deletion (pairwise deletion), and default values were used for other parameters. The phylogenetic tree was beautified by iTOL online tool.^9^

### 2.6 Fatty acid analysis

Strains TWZ-97, Mn4, SW8, S-28 and S-429 were cultured at 28°C in M4 medium with reciprocal shaking (170 rpm) for 7 days. Samples were taken every 24 h. Cells of the five strains were collected by centrifugation (8,000 rpm, 4°C, 10 min) and washed twice with sterile distilled water followed by lyophilization for 48 h. The dry cell weight was determined by the gravimetric method. Fatty acid methyl esters (FAME) were prepared as described previously with minor modification (Liu et al., 2014). Approximately 50 mg of lyophilized cells were weighed and mixed with 2 mL of 4% methanol sulfate (v/v) and 100 μL of nonadecanoic acid (1 mg/mL hexane), vortexed for 30 s and incubated in a water bath for 1 h at 80°C. After being cooled to room temperature, the resulting mixture was added with 1 mL of hexane and 1 mL ddH_2_O. The resulting upper hexane layer contain FAME was washed with 1 mL 5% NaCl (w/v) and 1 mL 2% KHCO_3_ (w/v) sequentially. The hexane layer was centrifuged, collected and dried with nitrogen gas. The FAME residues were resolved in 1 mL hexane and analyzed with a 7,890GC (Agilent Technologies, USA). Measurement conditions were as follows: DB-WAX column (60 m × 320 μm × 0.15 μm), hydrogen flame ionization detector (FID); nitrogen as the carrier gas, nitrogen flow rate of 1 mL/min; diversion mode, diversion ratio of 50:1; inlet temperature of 250°C; the temperature of the column box using the programmed temperature method, first set to 50°C for 1 min, then 25°C/min warming to 175°C, 3°C/min warming to 220°C and kept for 5 min, 2°C/min warming to 230°C and kept for 11 min. All the analyses were performed in triplicates.

## 3 Results

### 3.1 Genome assembly and annotation of *Aurantiochytrium* sp. TWZ-97

The genome of *Aurantiochytrium* sp. TWZ-97 was sequenced using Illumina and produced 64,184,996 bp clean data. GenomeScope estimated a genome size of 62,380,168 Mb with a heterozygosity of 0.98%. The final assembly yielded a genome of 62,493,101 bp, comprising 26 contigs, with an average contig length of 2,403,580.81 bp, and a GC content was 45.01% (Table 1). Genome completeness, assessed by BUSCO, showed 87% complete and single-copy BUSCO, indicating high assembly quality.

**Table 1:** Genomic characterization of *Aurantiochytrium* sp. TWZ-97.

Characteristics	Value
Genome size, Mb	62.49
Contigs	26
Average length of contigs, bp	2,403,580.81
Maximum length, bp	4,079,669
Minimum length, bp	1,119,645
N_50_, bp	2,583,946
N_90_, bp	1,678,149
GC content, mol%	45.01%

**Figure 1:**
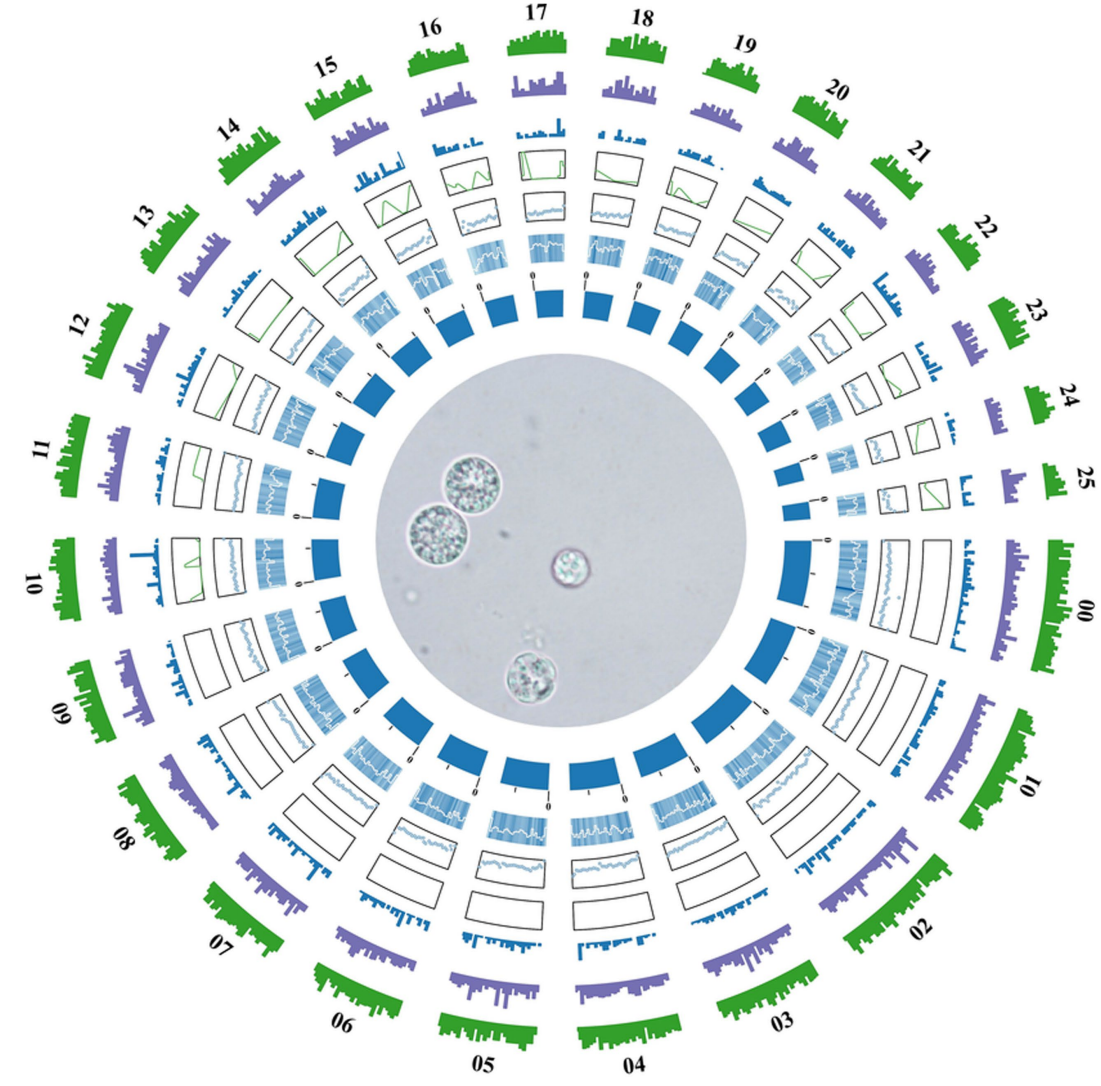
Genome circle diagram of *Aurantiochytrium* sp. TWZ-97. From the inner circle to the outer circle are the statistics of genome (sorted by length), GC content, GC skew, ncRNA density, TE density, repetitive sequence density, and gene density, respectively, according to a 10 kb window.

A total of 11,858 protein-coding genes were predicted through a combination of *ab initio* prediction, homology-based prediction, and transcriptomic-assisted prediction. For non-coding RNAs (ncRNAs), 552 tRNAs, 312 rRNAs and 9 snRNA genes were identified in the TWZ-97 genome (Supplementary Table 1). Repetitive elements accounted for 4,140,737 bp (6.63% of the genome sequence) with long terminal repeat sequences (LTRs) being the most abundant transposable elements, representing approximately 1.46% of the genome (Figure 1).

**Figure 2:**
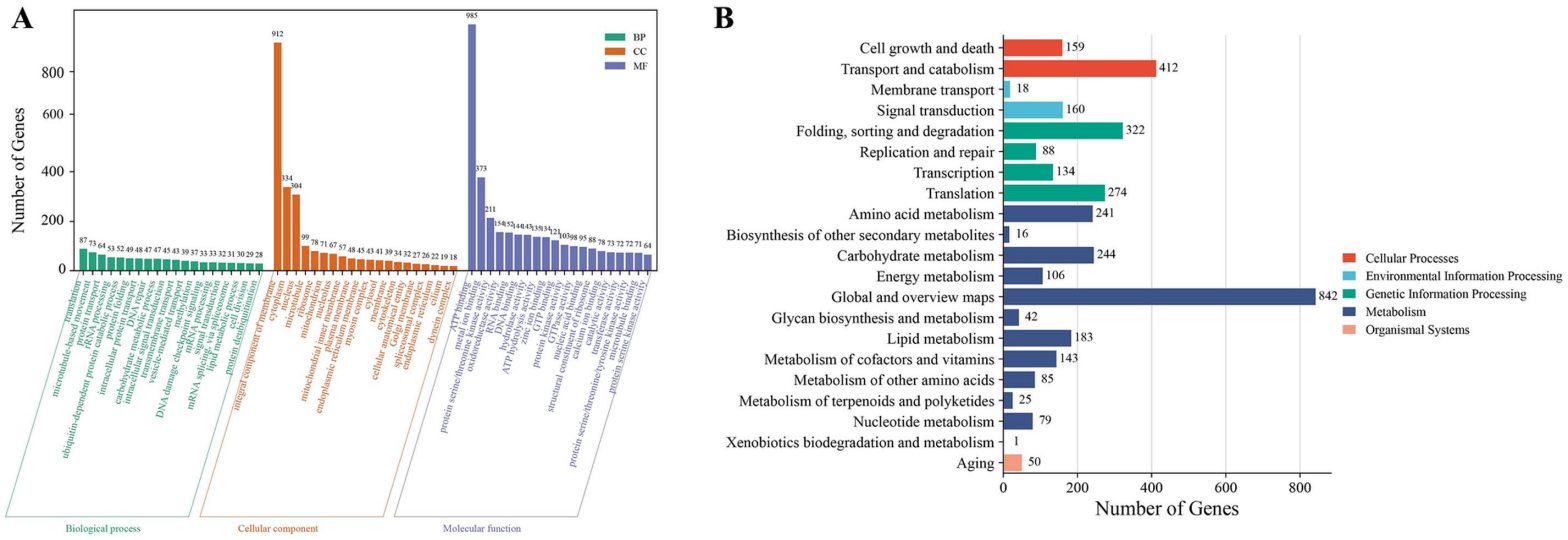
Results of gene function annotation of *Aurantiochytrium* sp. TWZ-97. **(A)** Distribution of functional annotations in the GO database. **(B)** Distribution of functional annotations in the KEGG database.

Functional annotation assigned 10,429 genes to at least one of the nine databases (KEGG, NR, UniProt, GO, KOG, Pfam, InterPro, RefSeq and TIGRFAM) (Supplementary Table 2). GO annotation results categorized 4,823 genes empty into three major categories: cellular components, molecular functions, and biological processes, accounting for 40.67% of the total number of TWZ-97 genes (Figure 2A). KEGG pathway analyses annotated 3,624 genes into five (cellular processes, environmental information processing, genetic information processing, metabolism, and organismal systems) in TWZ-97 (Figure 2B). Notably, 183 genes were associated with lipid metabolism, providing a valuable resource for future studies on fatty acid biosynthesis. Overall, these results demonstrate the successful acquisition of a high-quality genome assembly for *Aurantiochytrium* sp. TWZ-97.

### 3.2 Differences in genome structure

To explore genomic differences between thraustochytrid ecotypes, we analyzed the genomes of *Aurantiochytrium* sp. TWZ-97 (TWZ-97), *Botryochytrium* sp. S-28 (S-28), *Oblongichytrium* sp. S-429 (S-429), *Aurantiochytrium* sp. Mn4 (Mn4), and *Aurantiochytrium* sp. SW8 (SW8) (Supplementary Table 3). Notably, the genomes of Mn4 (65.69 Mb), SW8 (61.67 Mb), and TWZ-97 (62.49 Mb) were nearly twice the size of those in S-28 (36.22 Mb) and S-429 (43.24 Mb). However, the number of protein-coding genes of S-28 (18,696) and S-429 (18,058) outnumbered those of the three larger genomes such as TWZ-97 (11, 858), reflecting a significant difference in gene density (Song et al., 2018; Liu et al., 2023). In summary, the “anabolic” strains (TWZ-97, Mn4, and SW8) feature larger genomes with lower gene density, while the “catabolic” strains (S-28 and S-429) have smaller and gene-dense genomes.

Based on gene structure prediction, we further compared the genomic structure of TWZ-97, S-28 and S-429, which have significant differences in genome size (Table 2). The total number of repetitive sequences in TWZ-97 was approximately 2.8 times that of S-28 and 5.2 times that of S-429, and was dominated by LTR reverse transcriptional transposons and DNA transposons (1.46% each). The LTR elements can self-amplify via reverse transcription mechanism, driving genome expansion and structural variation, potentially enhancing adaptative capacity (Gervais and Shapiro, 2024; Huang et al., 2025). In contrast, repetitive sequences of S-28 genome were dominated by simple repeat sequences (2.64%) and low complexity repeat sequences (0.71%), while S-429 contained predominantly LTRs (0.54%) and simple repeat sequences (0.53%), which suggests that their genomes are more stable and less prone to mutation than TWZ-97. Simple repeat sequences are typically involved in gene regulation and chromatin conformation adjustment, indicating that these two strains may have an advantage in regulatory flexibility in specific functional domains (Kashi and King, 2006).

**Table 2:** Analysis of genomic repetitive sequences of S-28, S-429 and TWZ-97.

Repeat elements	S-28		S-429		TWZ-97	
	Repeat size (bp)	Percentage of the assembled genome	Repeat size (bp)	Percentage of the assembled genome	Repeat size (bp)	Percentage of the assembled genome
LTR	73,618	0.19	239,247	0.54	914,571	1.46
LTR/Gypsy	20,424	0.05	135,190	0.30	392,416	0.63
LTR/Copia	34,548	0.09	61,455	0.14	351,306	0.56
DNA	99,620	0.26	144,922	0.33	909,831	1.46
SINE	727	0.00	4,456	0.01	5,444	0.01
LINE	34,106	0.09	107,137	0.24	540,122	0.86
Satellite	11,632	0.03	17,229	0.04	50,732	0.08
Simple repeat	1,005,998	2.64	233,679	0.53	0	0.00
Low complexity	271,060	0.71	31,017	0.07	-	-
Other	10,878	0.03	24,040	0.05	364	0.00
Unknown	5,946	0.02	20,296	0.05	86,850	0.14
Total	1,490,994	3.91	788,933	1.78	4,140,737	6.63

Overall, the genome of the “anabolic” strain TWZ-97 is characterized by frequenct transposon activity, repetitive sequence expansion and gene loss, supporting rapid evolutionary potential. In contrast, the “catabolic” strains S-28 and S-429 exhibit genomic stability, enriched gene repertoires, and regulatory versatility—traits that underpin their capacity for environmental adaptability and the degradation of complex organic substrates.

### 3.3 Comparative genomics analysis

**Figure 3:**
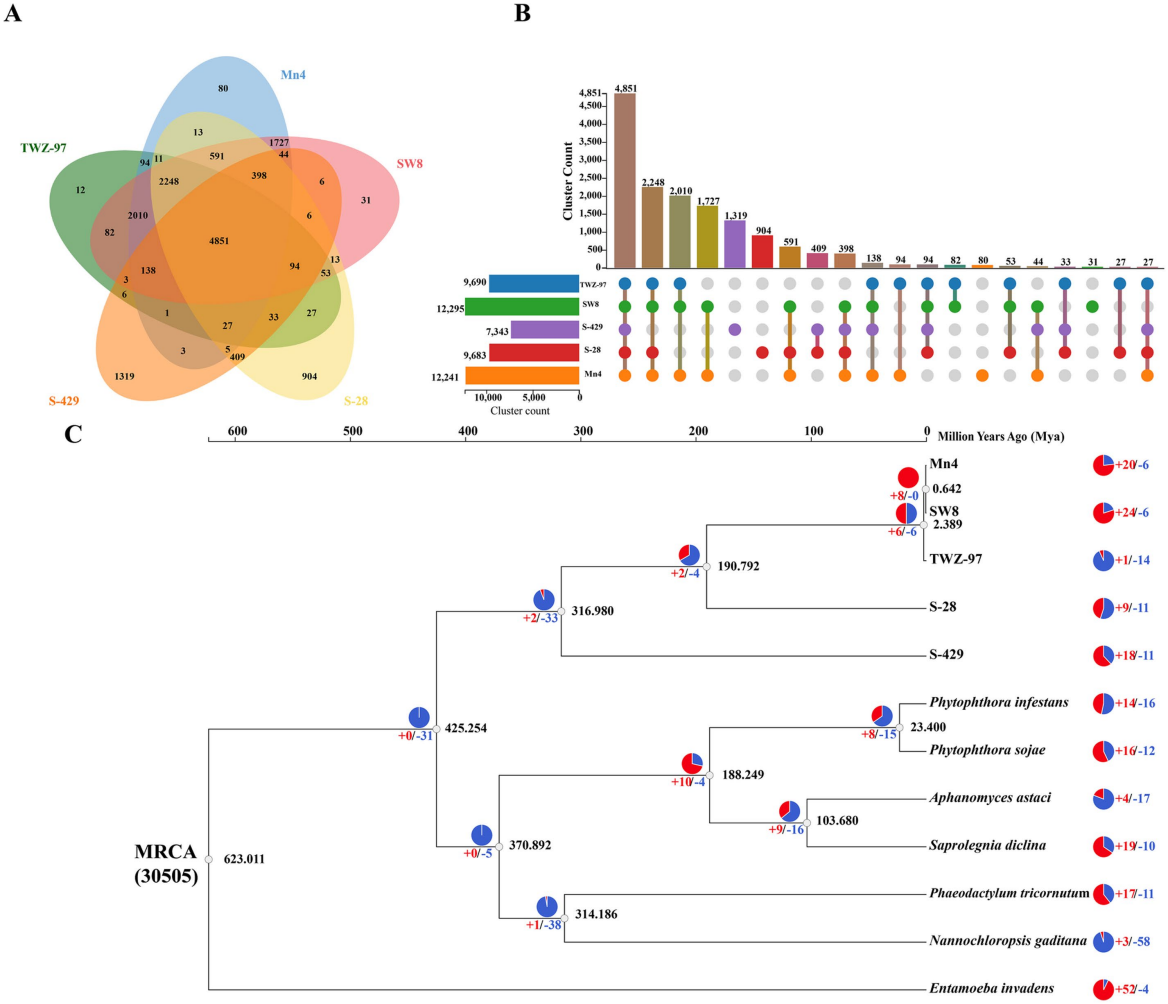
Analysis of gene families and phylogeny of TWZ-97 and other related genomes. **(A)** Venn diagram representing gene family clustering of TWZ-97 and four close relatives (Mn4, SW8, S-28, S-429). **(B)** The UpSet plot represents the distribution and number of gene family clusters in the five genomes. **(C)** Estimation of time to differentiation and expansion/contraction of gene families. Numbers labeled in red and blue represent contracted and expanded gene families, respectively.

In order to study the genomic evolutionary relationship between the two ecotype strains, we performed homologous gene identification and gene family clustering analysis on 5 genomes of thraustochytrids (TWZ-97, Mn4, SW8, S-28 and S-429) (Figures 3A,B). The results showed that the number of genes shared by the “anabolic” strains (TWZ-97, Mn4 and SW8) was 2010, and the number of genes shared by the “catabolic” strains (S-28 and S-429) was 409, indicating a much higher number of conserved orthologous genes among the “anabolic” strains. The “catabolic” strains had more unique orthologous genes (904 in S-28 and 1,319 in S-429). To further understand these differences, we conducted analysis on specific orthologous genes of each ecotype (Supplementary Tables 4, 5). Genes related to fatty acid anabolism (GO:0006631, GO:0006633, GO:0006629) were significantly enriched in the “anabolic” strain, while genes associated with hydrolytic enzyme activity (GO:0016787) exclusively present in the “catabolic” strains.

To explore the phylogenetic relationships, we conducted a phylogenetic tree based on the genomes of 12 species and estimated their divergence times, including five species of thraustochytrids and seven other protists (Figure 3C). The analysis showed that TWZ-97 diverged from Mn4 and SW8 at 2.389 Mya, suggesting that “anabolic” strains had a close evolutionary relationship. In contrast, the differentiation nodes of S-28 and S-429 occurred much earlier, indicating prolonged independent evolution. Lastly, gene family contraction and expansion analysis revealed no significant overall trend between the two ecotype. Moreover, 14 gene families were significantly contracted but 1 gene family was significantly expanded in the TWZ-97 genome, with significantly fewer gene family expansions and more gene family contractions than in other thraustochytrids.

### 3.4 Identification of gene families for fatty acid desaturase (FAD) and polyketide synthase (PKS)

In thraustochytrids, unsaturated fatty acids are synthesized via two main pathways: the FAS pathway introduces double bonds through fatty acid desaturase (FAD), while, the PKS pathway directly produce long-chain PUFAs using a modular multi-enzyme complex. Thus, FAD and PKS genes play key roles in fatty acid biosynthesis. To explore metabolic differences between the two ecotype strains, we identified 45 FAD genes and 20 PKS genes in the TWZ-97, S-28 and S-429 genomes, which were highly differentiated. They were named based on genus name and their genomic position information (AuFAD, AuPKS, BoFAD, BoPKS, ObFAD, ObPKS) (Supplementary Tables 6, 7). Detailed information on gene ID, gene location and size, protein length (aa), molecular weight (MW), theoretical isoelectric point (pI) and subcellular is provided (Supplementary Tables 6, 7). FAD genes were numerous and dispersed across the three genomes without notable gene clustering. In contrast, PKS genes were fewer, longer, and tended to cluster in specific genomic region, featuring more complex structural domains (Marchan et al., 2018; Morabito et al., 2019). The total number of FAD and PKS genes in the “catabolic” strains (S-28 and S-429) far exceeded that of the “anabolic” strain (TWZ-97), indicating a more functionally enriched gene set in the “catabolic” ecotypes. Notably, S-429 harbored 15 PKS genes, compared to just 3 in TWZ-97 and 2 in S-28, suggesting unique capabilities in secondary metabolism and environmental adaptation, such as multi-pathway product synthesis or efficient substrate catabolism.

**Figure 4:**
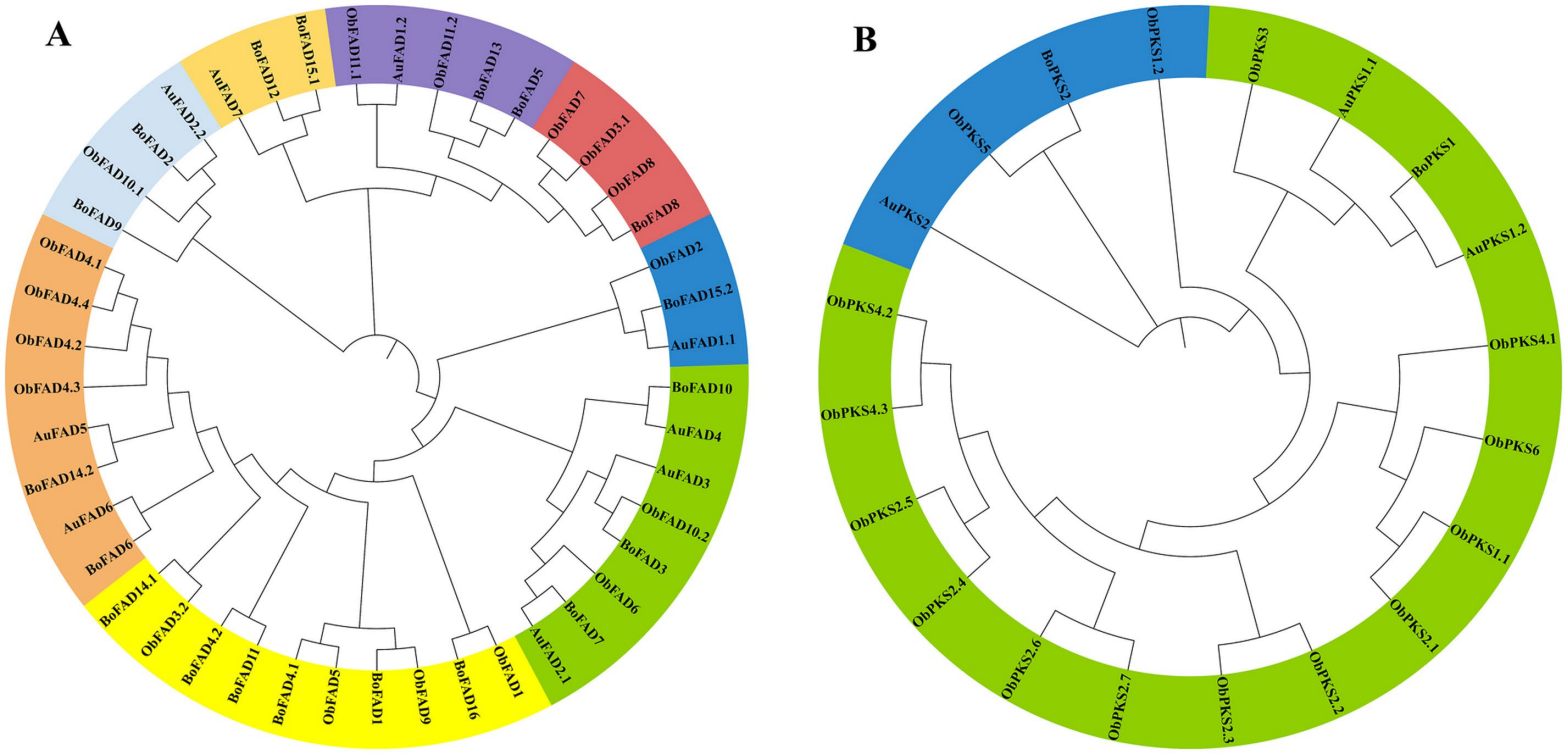
Phylogenetic tree for TWZ-97, S-28 and S-429 FAD genes **(A)** and PKS genes **(B)**. The FAD genes and PKS genes from TWZ-97 (*Aurantiochytrium*, AuFADs, AuPKSs), S-28 (*Botryochytrium*, BoFADs, BoPKSs) and S-429 (*Oblongichytrium*, ObFADs, ObPKSs) were firstly aligned using the ClustalW, and the phylogenetic tree was then constructed using MEGAX by the neighbor-joining method. A total of 1,000 bootstrap replications were applied.

To illustrate the phylogenetic relationship, neighbor-joining trees were constructed for FAD genes and PKS genes from TWZ-97, S-28, and S-429 (Figure 4). Phylogenetic analysis showed that the FAD genes and PKS genes can be roughly divided into eight and two subfamilies, respectively. The evolutionary relationship patterns were broadly similar between the ecotypes, though the FAD gene family was more abundant and showed greater phylogenetic complexity.

**Figure 5:**
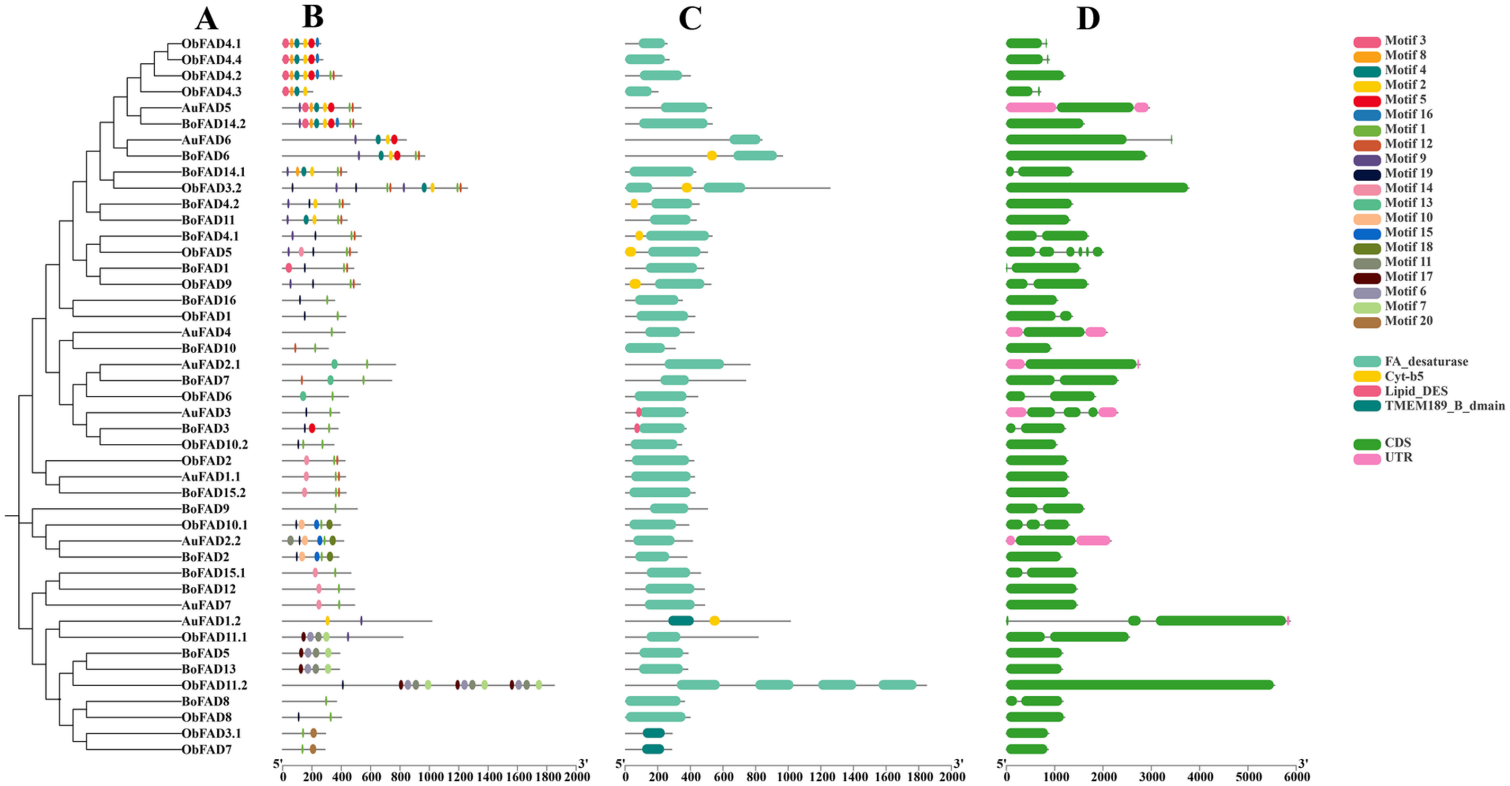
Gene structure of FADs in TWZ-97, S-28 and S-429. **(A)** Phylogenetic tree for FAD genes; **(B)** Various colors in the ellipse-shaped are representing the conserved motifs; **(C)** The conserved domain is represented in different colors; **(D)** CDS is displayed in green color, UTR is displayed in green color, whereas distinct introns are displayed in gray lines.

**Figure 6:**
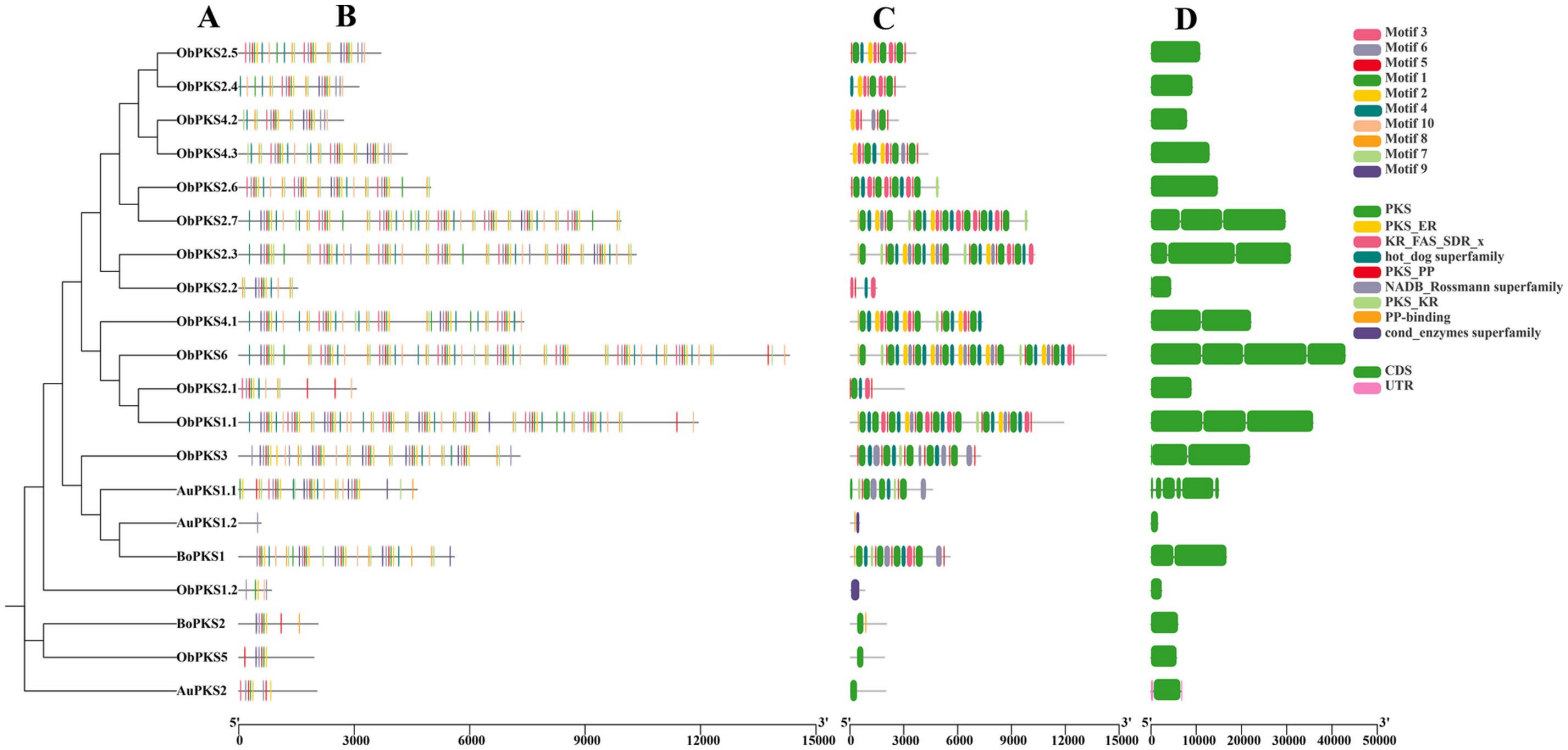
Gene structure of PKSs in TWZ-97, S-28 and S-429. **(A)** Phylogenetic tree for PKS genes; **(B)** Various colors in the box-shaped are representing the conserved motifs; **(C)** The conserved domain is represented in different colors; **(D)** CDS is displayed in green color, UTR is displayed in green color, whereas distinct introns are displayed in gray lines.

The gene structures, motifs, and conserved structural domains of the FAD and PKS genes were further analyzed (Figures 5, 6). All FAD genes had the FA_desaturase structural domain except AuFAD1.2, ObFAD3.1 and ObFAD7, which had the TMEM189_B_dmain structural domain. All PKS genes except AuPKS1.2, ObPKS1.2 and ObPKS2.2 contained PKS structural domains, and the number of PKS structural domains varied, from a minimum of one PKS structural domain to a maximum of eight PKS structural domains. The variety and number of conserved structural domains identified for PKS genes were significantly greater than those for FAD genes. The vast majority of FAD genes contained 1–2 exons, indicating structural conservation and functional stability. In contrast, PKS genes had a higher number of exons, with 1–6 exons, indicating a more complex structure. Members of the same subfamily showed similar exon/intron distribution patterns. Motif analysis using MEME identified 20 motifs in FAD genes and 10 in PKS genes. FAD genes exhibited a length-independent motif pattern, where essential catalytic motifs were maintained while redundant regions varied. PKS genes, however, showed a modular motif distribution, with motif numbers positively correlated with gene length, reflecting the presence of collaborative enzymatic domains like acyl carrier protein (ACP) binding sites. Within both FAD and PKS families, motifs and exon/intron structures were largely conserved within subfamilies, indicating shared biological functions, while distinct motifs characterized different subgroups. While the number, domain architecture, and coding sequence lengths of FAD genes were comparable between “anabolic” and “catabolic” strains, PKS genes in the “catabolic” strains exhibited significantly more motifs, domains, and longer coding sequences than in TWZ-97. This points to a type-specific divergence in PKS gene complexity between the two ecotypes.

### 3.5 Fatty acid production capacity of different ecotypes of thraustochytrids

**Figure 7:**
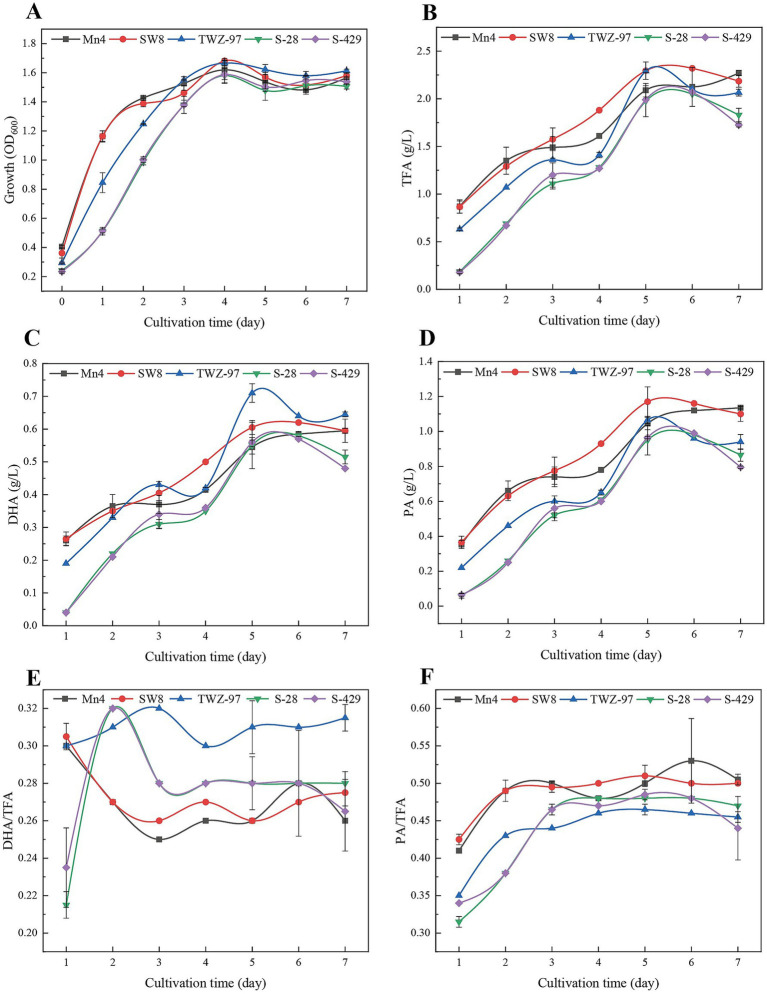
Time-dependent determination of changes in growth status **(A)**, total fatty acid production (TFA) **(B)**, DHA production **(C)**, PA production **(D)**, DHA-to-TFA production ratio **(E)** and PA-to-TFA production ratio **(F)** of thraustochytrids (TWZ-97, Mn4, SW8, S-28 and S-429).

To assess differences in fatty acid production between the two ecotype strains, we determined the growth patterns and fatty acid yield of TWZ-97, Mn4, SW8, S-28, and S-429 (Figure 7). The strains exhibited a biphasic growth pattern: during the early phase (0–2 days), the “anabolic” strains (TWZ-97, Mn4, and SW8) showed significantly faster biomass accumulation than the “catabolic” strains (S-28 and S-429). By days 3–7, all strains entered a stable growth phase. Total fatty acid (TFA) synthesis showed significant differences among the strains. On day 5, TWZ-97, Mn4, and SW8 reached TFA concentrations of 2.29, 2.09, and 2.29 g/L, respectively, outperforming S-28 (1.98 g/L) and S-429 (1.99 g/L). TWZ-97 also exhibited a clear advantage in docosahexaenoic acid (DHA) production, displaying an S-shaped yield curve with a rapid increase to 0.14 g/L/day by day 2 and peaking at 0.71 g/L on day 5 — substantially higher than Mn4 (0.55 g/L). For saturated fatty acids, SW8 excelled in palmitic acid (PA) synthesis, achieving 1.17 g/L by day 3 — a 23.2% increase over S-28 (0.95 g/L). The PA/TFA ratio remained consistent across strains, ranging from 0.47 to 0.51, while TWZ-97 reached the highest DHA/TFA ratio at 0.31. In summary, while both ecotype groups are capable of fatty acid production, the “anabolic” strains demonstrate superior yields and a greater capacity for high-value unsaturated fatty acid synthesis.

## 4 Discussion

Thraustochytrids have attracted significant attention for their high fatty acid production potential. In this study, we present a high-quality genome assembly of *Aurantiochytrium* sp. TWZ-97. Assembly metrics, including N50, N90, and BUSCO scores, confirmed its high continuity and integrity. The TWZ-97 genome is 62.49 Mb, comparable to Mn4 (65.69 Mb) and SW8 (61.67 Mb) (Song et al., 2018), and larger than S-28 (36.22 Mb) and S-429 (43.24 Mb) (Liu et al., 2023). Other thraustochytrid genomes similarly fall into two size categories: larger genomes include *Schizochytrium* sp. TIO01 (64.00 Mb) (Hu et al., 2020), *Schizochytrium limacinum* SR21 (63.00 Mb) (Liang et al., 2020), *Thraustochytriidae* sp. SZU445 (63.55 Mb) (Zhu et al., 2020), and *Aurantiochytrium* sp. SW1 (60.89 Mb) (Prabhakaran et al., 2022); smaller genomes include *Thraustochytrium* sp. 26,185 (38.60 Mb) (Zhao et al., 2016), *Schizochytrium* sp. CCTCC M209059 (39.09 Mb) (Ji et al., 2015), *Schizochytrium* sp. S31 (42.99 Mb) (Chang et al., 2021), and *Aurantiochytrium* sp. T66 (43.00 Mb) (Liu et al., 2016).

Genome size in thraustochytrids is shaped by factors such as gene duplications, transposonable elements, gene loss, horizontal gene transfer, and genomic rearrangements. While genome size does not directly reflect organismal complexity, it often mirrors the evolutionary history and ecological strategies. The larger genome, high transposon content and low gene density in the “anabolic” strain TWZ-97 suggest a “plasticity-first” strategy (Levis and Pfennig, 2016), favoring environmental adaptability through transposon-mediated genome reorganization at the cost of some gene loss. In contrast, “catabolic” strains have smaller, compact genomes that reduce DNA replication energy costs, supporting rapid growth and efficient catabolism via high secretion of degradative enzymes (Dong et al., 2024). Their high gene density supports complex organic matter degradation (e.g., secretion of CAZymes and lipases) and genome compactness (reduced non-coding regions) for metabolic efficiency (Leushkin et al., 2013; Fernández et al., 2024). The abundance of secreted proteins in S-28 and S-429 highlights their roles as efficient decomposers, while TWZ-97 likely relies on alternative metabolic pathways. These findings provide new insights into genome size variation, evolutionary dynamics, and gene structure diversification in thraustochytrids.

Species relationships and divergence times is fundamental to evolutionary biology. In this study, phylogenetic analysis and gene family expansion/contraction profiling revealed that TWZ-97 is the most closely related to Mn4 and SW8, consistent with prior studies (Song et al., 2018; Liu et al., 2023). The close genetic relationship among TWZ-97, Mn4, and SW8 (short divergence time and many shared gene families) supports their monophyletic origin. S-28 and S-429 represent earlier lineages with different adaptation strategies. The gene family contraction in “anabolic” strains like TWZ-97 reflects specialization, while the moderate expansion in homozygous Mn4 and SW8 indicates enhanced functional diversity and strategic flexibility. Among the “catabolic” strains, the expansion of S-429 likely reflect long-term independent evolution, while S-28 retained a more balanced, streamlined genome, representing a mainstream catabolic strategy.

In recent years, the high lipid content of thraustochytrids has gained increasing attention due to their potential multiple benefits to human health. In this study, we identified key genes and pathways involved in fatty acid biosynthesis in TWZ-97. Comparison of the genomes of the different ecotypes of strains showed that the “catabolic” strains (S-29, S-429) had smaller genomes, but had a significant expansion of the FAD/PKS gene family, which contrasted with the expansion of the genome (62.5 Mb) and contraction of the metabolic genes in the “anabolic” strains (TWZ-97). This apparent contradiction may reflect the differentiated adaptation strategies of the two ecotype strains to the heterogeneity of marine habitats. At the level of genome structure, the compact, gene-dense, and low transposon genomes of “catabolic” strains impose strong selection pressure to retain functional genes (Condic et al., 2024). Their FAD/PKS genes often form tandem clusters via duplication, likely safeguarding gene function through physical proximity. Conversely, the “anabolic” strains, characterized by genome expansion driven by transposons and non-coding sequence accumulation, face relaxed selective pressure on metabolic genes, promoting gradual loss of redundancy (Gervais and Shapiro, 2024; Li et al., 2025). Functionally, “catabolic” strains must rapidly metabolize complex, dynamic organic substrates in marine debris. Their expanded, diversified FAD/PKS gene clusters likely enable both the breakdown of lipid-rich organic matter and the re-esterification or synthesis of storage lipids via modular PKS pathways. This “coupled degradation-synthesis” mechanism ensures metabolic homeostasis in fluctuating carbon environments, while TWZ-97 and other “anabolic” strains pursue a specialization strategy geared toward high-value fatty acid biosynthesis under more stable conditions.

## 5 Conclusion

A comparative genomic analysis of high-quality thraustochytrid genomes have revealed a significant divergence in genome structure, phylogeny, and metabolic function between “anabolic” and “catabolic” strains. The “anabolic” strains (TWZ-97, Mn4, and SW8) possess large genomes (60–65 Mb), lower gene density, and extensive, repetitive sequence expansions, notably with transposon activation in TWZ-97. This finding suggests the potential for rapid evolution in the “anabolic” strains. In contrast, the “catabolic” strains (S-28 and S-429) have smaller genomes (<45 Mb) and exhibit higher gene density, indicating efficient catabolism supported by stable repetitive sequences and expansive gene pools. Phylogenetic analysis revealed that the “anabolic” strains have recently evolved into distinct monophyletic groups, whereas the “catabolic” strains diverged from their ancestors over 190 Mya, resulting prolonged and independent adaptation. Unique gene families in the “catabolic” strains further highlight their distinct evolutionary trajectories. Functionally, the “anabolic” strains are enriched in fatty acid synthase genes but lacked hydrolytic enzymes, whereas the “catabolic” strains specialized in hydrolytic enzymes while retaining partial fatty acid synthesis capacity. Additionally, type-specific differentiation of the PKS genes was observed between the two ecotypes. These divergent traits offer complementary biotechnological applications: “anabolic” strains, TWZ-97 (high DHA prodocution) and SW8 (high PA production)for lipid engineering, while “catabolic” strains hold promise for environmental remediation through the degradation of organic waste via their hydrolase repertoire.

## Data Availability

The original contributions presented in the study are publicly available. This data can be found at: https://www.ncbi.nlm.nih.gov/, accession number: PRJNA1247126.

## Footnotes

^1^ https://orthovenn3.bioinfotoolkits.net/home

^2^ http://pfam.xfam.org/

^3^ http://smart.embl-heidelberg.de/

^4^ https://services.healthtech.dtu.dk/services/DeepTMHMM-1.0/

^5^ https://services.healthtech.dtu.dk/services/SignalP-5.0/

^6^ http://cello.life.nctu.edu.tw/

^7^ https://meme-suite.org/meme/tools/meme

^8^ https://www.ncbi.nlm.nih.gov/Structure/bwrpsb/bwrpsb.cgi

^9^ https://itol.embl.de/login.cgi
