# Supplementary material for: Genomic evolution and ecotype divergence in thraustochytrids: insights from comparative genomics and phylogenomics
Source: Front Microbiol. 2025 Jun 30;16:1608951. doi: 10.3389/fmicb.2025.1608951 (PMC12258536; doi:10.3389/fmicb.2025.1608951)
Supplement: Supplementary file 1 [file Data_Sheet_1.docx]

Supplementary Material

| Type |  | Copy | Average length (bp) | Total length (bp) | % of genome |
| --- | --- | --- | --- | --- | --- |
| miRNA |  | 0 | 0 | 0 | 0 |
| tRNA |  | 552 | 79 | 43339 | 0.06935 |
| rRNA | rRNA | 312 | 372 | 115970 | 0.185572 |
|  | 18S | 91 | 954 | 86799 | 0.138894 |
|  | 28S | 0 | 0 | 0 | 0 |
|  | 5.8S | 90 | 151 | 13590 | 0.021746 |
|  | 5S | 131 | 119 | 15581 | 0.024932 |
| snRNA | snRNA | 9 | 163 | 1467 | 0.002347 |
|  | CD-box | 6 | 171 | 1024 | 0.001639 |
|  | HACA-box | 0 | 0 | 0 | 0 |
|  | splicing | 3 | 148 | 443 | 0.000709 |
|  | scaRNA | 0 | 0 | 0 | 0 |

**Supplementary Table 1.** Non-coding RNA annotation results.

**Supplementary Table 2.** Functional annotation results of *Aurantiochytrium* sp. TWZ-97.

| Item | Count | Percentage |
| --- | --- | --- |
| All | 11,858 | 100.00% |
| Annotation | 10,429 | 87.95% |
| KEGG | 2,581 | 21.77% |
| Pathway | 2,099 | 17.70% |
| NR | 6,686 | 56.38% |
| UniProt | 6,101 | 51.45% |
| GO | 4,823 | 40.67% |
| KOG | 2,270 | 19.14% |
| Pfam | 7,105 | 59.92% |
| InterPro | 10,370 | 87.45% |
| RefSeq | 5,679 | 47.89% |
| TIGRFAM | 2,286 | 19.28% |

**Supplementary Table 3.** Differences in genomic structure of S-28, S-429, Mn4, SW8 and TWZ-97.

|  | S-28 | S-429 | Mn4 | SW8 | TWZ-97 |
| --- | --- | --- | --- | --- | --- |
| Scaffolds, no. | 44 | 45 | 1,611 | 1,202 | 26 |
| Genome size, Mb | 36.22 | 43.24 | 65.69 | 61.67 | 62.49 |
| Maximum length, bp | 2,173,176 | 2,291,525 | 667,038 | 659,658 | 4,079,669 |
| N50, bp | 869,909 | 1,366,673 | 153,854 | 127,831 | 2,583,946 |
| N90, bp | 597,294 | 761,285 | 14,564 | 22,659 | 1,678,149 |
| GC content, mol% | 47.47 | 43.3 | 44.93 | 45.11 | 45.01 |
| Rate of N, % | 0 | 0 | 0 | 0 | 0 |
| No. of predicted CDSs | 18,696 | 18,058 | 17,887 | 16,574 | 11,858 |
| No. of secreted proteins | 1,840 | 1,483 | 297 | 252 | 248 |
| No. of genes annotated with: | | | | | |
| COG database | 5,828 | 5,995 | 5,295 | 5,229 | 2,270 |
| KEGG database | 3,391 | 3,698 | 1,817 | 1,839 | 2,581 |

**Supplementary Table 4.** GO enrichment results for fatty acid biosynthesis metabolism genes.

| SampleGroup | GO | Description | Count | p-value |
| --- | --- | --- | --- | --- |
| Mn4&SW8&TWZ-97 | [GO:0006631](http://amigo.geneontology.org/amigo/term/GO:0006631) | fatty acid metabolic process | 3 | 8.59E-14 |
| Mn4&SW8&TWZ-97 | [GO:0006665](http://amigo.geneontology.org/amigo/term/GO:0006665) | sphingolipid metabolic process | 3 | 1.65E-08 |
| Mn4&SW8&TWZ-97 | [GO:0006633](http://amigo.geneontology.org/amigo/term/GO:0006633) | fatty acid biosynthetic process | 6 | 5.20E-08 |
| Mn4&SW8&TWZ-97 | [GO:0006629](http://amigo.geneontology.org/amigo/term/GO:0006629) | lipid metabolic process | 3 | 3.05E-07 |
| Mn4&SW8&TWZ-97 | [GO:0008654](http://amigo.geneontology.org/amigo/term/GO:0008654) | phospholipid biosynthetic process | 7 | 3.89E-06 |
| Mn4&SW8&TWZ-97 | [GO:0008289](http://amigo.geneontology.org/amigo/term/GO:0008289) | lipid binding | 3 | 1.60E-05 |
| Mn4&SW8&TWZ-97 | [GO:0019433](http://amigo.geneontology.org/amigo/term/GO:0019433) | triglyceride catabolic process | 3 | 0.000405033000 |
| Mn4&SW8&TWZ-97 | [GO:0008610](http://amigo.geneontology.org/amigo/term/GO:0008610) | lipid biosynthetic process | 3 | 0.000679860000 |
| Mn4&SW8&TWZ-97 | [GO:0006027](http://amigo.geneontology.org/amigo/term/GO:0006027) | glycosaminoglycan catabolic process | 2 | 0.002933516000 |
| S-28&S-429 | [GO:0016126](http://amigo.geneontology.org/amigo/term/GO:0016126) | sterol biosynthetic process | 2 | 8.04E-07 |
| S-28&S-429 | [GO:0006636](http://amigo.geneontology.org/amigo/term/GO:0006636) | unsaturated fatty acid biosynthetic process | 9 | 7.11E-05 |
| S-28&S-429 | [GO:0006631](http://amigo.geneontology.org/amigo/term/GO:0006631) | fatty acid metabolic process | 3 | 0.000426439000 |
| S-28&S-429 | [GO:0006869](http://amigo.geneontology.org/amigo/term/GO:0006869) | lipid transport | 3 | 0.007254105000 |
| S-28&S-429 | [GO:0006629](http://amigo.geneontology.org/amigo/term/GO:0006629) | lipid metabolic process | 2 | 0.010031847000 |

**Supplementary Table 5.** GO enrichment results for hydrolase genes.

| SampleGroup | GO | Description | Count | p-value |
| --- | --- | --- | --- | --- |
| S-28&S-429 | [GO:0016787](http://amigo.geneontology.org/amigo/term/GO:0016787) | hydrolase activity | 2 | 0.000404831 |
| S-28&S-429 | [GO:0004518](http://amigo.geneontology.org/amigo/term/GO:0004518) | nuclease activity | 4 | 0.001794764 |
| S-28&S-429 | [GO:0005886](http://amigo.geneontology.org/amigo/term/GO:0005886) | plasma membrane | 7 | 0.000130386 |

**Supplementary Table 6.** Basic information for the FAD gene family members of S-28, S-429 and TWZ-97.

| Gene ID | Contig | Number of Amino Acid (bp) | Molecular Weight (Da) | Theoretical pI | Instability Index | Aliphatic Index | Grand Average of Hydropathicity | Transmembrane domain （DeepTMHMM） | Signal peptide | Subcellular localization |
| --- | --- | --- | --- | --- | --- | --- | --- | --- | --- | --- |
| AuFAD1.1 | 1 | 428 | 49360.74 | 6.08 | 33.15 | 87.73 | -0.091 | 4 | NO | endomembrane system |
| AuFAD1.2 | 1 | 1016 | 113946.53 | 8.35 | 45.87 | 88.34 | -0.135 | 4 | NO | cytoplasm |
| AuFAD2.1 | 2 | 769 | 87786.58 | 6.75 | 45.57 | 89.71 | -0.112 | 4 | NO | endomembrane system |
| AuFAD2.2 | 2 | 416 | 46916.56 | 9.21 | 36.02 | 74.78 | -0.242 | 4 | NO | extracellular space |
| AuFAD3 | 7 | 388 | 44674.24 | 6.57 | 43.02 | 85.23 | -0.186 | 4 | NO | endomembrane system |
| AuFAD4 | 8 | 426 | 50421.92 | 6.93 | 47.24 | 73.69 | -0.337 | 4 | NO | endomembrane system |
| AuFAD5 | 13 | 533 | 62543.14 | 7.35 | 40.58 | 80.86 | -0.106 | 7 | NO | endomembrane system |
| AuFAD6 | 15 | 842 | 93825.69 | 8.19 | 40.04 | 101.44 | 0.267 | 15 | NO | endomembrane system |
| AuFAD7 | 19 | 490 | 55116.41 | 9.58 | 39.69 | 91.63 | -0.109 | 4 | NO | endomembrane system |
| BoFAD1 | 5 | 484 | 55110.94 | 9.03 | 28.81 | 80 | -0.149 | 4 | NO | endomembrane system |
| BoFAD10 | 38 | 311 | 36873.91 | 8.88 | 47.93 | 88.04 | 0.067 | 4 | NO | endomembrane system |
| BoFAD11 | 39 | 439 | 49968.7 | 9.05 | 30.68 | 86.86 | -0.117 | 4 | NO | endomembrane system |
| BoFAD12 | 40 | 489 | 56128.72 | 9.64 | 34.63 | 82.39 | -0.296 | 4 | NO | organelle membrane |
| BoFAD13 | 42 | 387 | 44371.22 | 8.94 | 35.7 | 84.42 | -0.102 | 5 | NO | endomembrane system |
| BoFAD14.1 | 45 | 437 | 50305.85 | 6.15 | 50.36 | 90.34 | -0.053 | 4 | NO | endomembrane system |
| BoFAD14.2 | 45 | 537 | 61916.41 | 7.75 | 40.72 | 88.25 | -0.073 | 6 | NO | endomembrane system |
| BoFAD15.1 | 46 | 465 | 53082.26 | 9.62 | 35.08 | 85.78 | -0.268 | 2 | NO | endomembrane system |
| BoFAD15.2 | 46 | 432 | 50531.94 | 6.81 | 32.22 | 84.84 | -0.178 | 4 | NO | endomembrane system |
| BoFAD16 | 47 | 354 | 41562.11 | 7.75 | 37.42 | 82.57 | 0.016 | 4 | NO | endomembrane system |
| BoFAD2 | 8 | 382 | 44487.03 | 8.24 | 31.14 | 79.32 | -0.236 | 4 | NO | endomembrane system |
| BoFAD3 | 18 | 377 | 43675.24 | 8.4 | 37.77 | 77.16 | -0.271 | 4 | NO | endomembrane system |
| BoFAD4.1 | 19 | 536 | 60910.69 | 6.7 | 29.27 | 82.93 | -0.182 | 6 | NO | endomembrane system |
| BoFAD4.2 | 19 | 457 | 52828.83 | 9.21 | 33.18 | 75.32 | -0.263 | 6 | NO | endomembrane system |
| BoFAD5 | 26 | 389 | 44356.01 | 9.09 | 30.47 | 82.75 | -0.143 | 5 | NO | organelle membrane |
| BoFAD6 | 29 | 968 | 108959.54 | 5.74 | 46.27 | 101.15 | 0.21 | 16 | NO | endomembrane system |
| BoFAD7 | 32 | 742 | 85087.49 | 6.28 | 46.72 | 88.99 | -0.09 | 6 | NO | endomembrane system |
| BoFAD8 | 34 | 367 | 42134.7 | 8.82 | 38.78 | 87.3 | -0.132 | 6 | NO | endomembrane system |
| BoFAD9 | 37 | 508 | 58355.27 | 8.82 | 34.04 | 88.74 | 0.014 | 7 | NO | endomembrane system |
| ObFAD1 | 5 | 431 | 49804.41 | 8.81 | 56.86 | 86.22 | -0.11 | 5 | NO | endomembrane system |
| ObFAD10.1 | 25 | 393 | 45148.88 | 8.42 | 39.67 | 85.85 | -0.162 | 4 | NO | endomembrane system |
| ObFAD10.2 | 25 | 349 | 40619.7 | 7.34 | 35.2 | 77.97 | -0.11 | 4 | NO | endomembrane system |
| ObFAD11.1 | 38 | 819 | 91749.2 | 8.74 | 40.81 | 76.84 | -0.401 | 2 | NO | plasma membrane |
| ObFAD11.2 | 38 | 1850 | 210688.68 | 8.93 | 40.59 | 88.65 | -0.057 | 17 | NO | endomembrane system |
| ObFAD2 | 7 | 424 | 48899.36 | 6.85 | 36.56 | 86.49 | -0.207 | 4 | NO | endomembrane system |
| ObFAD3.1 | 10 | 292 | 32505.87 | 8.47 | 39.59 | 98.49 | 0.26 | 4 | NO | endomembrane system |
| ObFAD3.2 | 10 | 1259 | 141955.18 | 7.21 | 42.3 | 85.2 | -0.055 | 14 | NO | endomembrane system |
| ObFAD4.1 | 11 | 260 | 30422.14 | 7.71 | 58.76 | 90.35 | -0.003 | 5 | NO | endomembrane system |
| ObFAD4.2 | 11 | 403 | 47386.69 | 8.11 | 50.96 | 83.23 | -0.162 | 6 | NO | endomembrane system |
| ObFAD4.3 | 11 | 205 | 23591.06 | 7.73 | 55.44 | 83.66 | -0.143 | 2 | NO | endomembrane system |
| ObFAD4.4 | 11 | 273 | 31985.88 | 8.58 | 59.37 | 87.84 | -0.064 | 5 | NO | endomembrane system |
| ObFAD5 | 15 | 508 | 57677.24 | 8.67 | 28.46 | 88.7 | -0.033 | 6 | NO | endomembrane system |
| ObFAD6 | 16 | 448 | 51331.49 | 5.68 | 37.6 | 90.54 | -0.09 | 4 | NO | endomembrane system |
| ObFAD7 | 20 | 288 | 32103.42 | 8.47 | 38.9 | 98.51 | 0.258 | 4 | NO | endomembrane system |
| ObFAD8 | 23 | 401 | 46108.42 | 9.18 | 37.96 | 88.25 | -0.062 | 6 | NO | endomembrane system |
| ObFAD9 | 24 | 529 | 59000.29 | 7.39 | 36.12 | 81.17 | -0.155 | 4 | NO | endomembrane system |

**Supplementary Table 7.** Basic information for the PKS gene family members of S-28, S-429 and TWZ-97.

| Gene ID | Contig | Number of Amino Acid (bp) | Molecular Weight (Da) | Theoretical pI | Instability Index | Aliphatic Index | Grand Average of Hydropathicity | Transmembrane domain （DeepTMHMM） | Signal peptide | Subcellular localization |
| --- | --- | --- | --- | --- | --- | --- | --- | --- | --- | --- |
| AuPKS1.2 | 12 | 575 | 63275.76 | 4.76 | 48 | 87.37 | -0.221 | 0 | NO | Nuclear |
| ObPKS1.2 | 1 | 849 | 91919.86 | 5.51 | 37.97 | 79.99 | -0.218 | 0 | NO | Cytoplasmic |
| ObPKS2.2 | 4 | 1526 | 167401.95 | 4.99 | 43.27 | 86.97 | -0.172 | 0 | NO | Chloroplast |
| ObPKS5 | 13 | 1946 | 214045.56 | 5.38 | 38.65 | 84.57 | -0.215 | 0 | NO | Cytoplasmic |
| AuPKS2 | 15 | 2027 | 220934.36 | 6.49 | 39.73 | 84.16 | -0.223 | 0 | NO | Cytoplasmic |
| BoPKS2 | 47 | 2053 | 224918.91 | 6.1 | 38.91 | 87.32 | -0.169 | 0 | NO | Cytoplasmic |
| ObPKS4.2 | 11 | 2719 | 296029.35 | 5.12 | 40.16 | 90.28 | -0.098 | 0 | NO | Cytoplasmic |
| ObPKS2.1 | 4 | 3051 | 336919.66 | 5.21 | 42.91 | 96.06 | 0.023 | 6 | NO | PlasmaMembrane |
| ObPKS2.4 | 4 | 3118 | 341197.07 | 5.16 | 40.56 | 88.94 | -0.138 | 0 | NO | Cytoplasmic |
| ObPKS2.5 | 4 | 3688 | 401972.96 | 5.13 | 40.78 | 88.82 | -0.122 | 0 | NO | Cytoplasmic |
| ObPKS4.3 | 11 | 4376 | 476789.88 | 5.03 | 39.66 | 89.91 | -0.103 | 0 | NO | Cytoplasmic |
| AuPKS1.1 | 12 | 4633 | 506365.75 | 5.87 | 43.52 | 90.6 | -0.114 | 0 | NO | PlasmaMembrane |
| ObPKS2.6 | 4 | 4983 | 543332.1 | 4.88 | 44.43 | 89.18 | -0.122 | 0 | NO | PlasmaMembrane |
| BoPKS1 | 3 | 5600 | 608858.27 | 5.48 | 40.98 | 90.68 | -0.063 | 0 | NO | Nuclear |
| ObPKS3 | 9 | 7306 | 800002.96 | 5.64 | 39.38 | 85.82 | -0.181 | 0 | NO | PlasmaMembrane |
| ObPKS4.1 | 11 | 7406 | 808073.38 | 5.17 | 39.94 | 90.28 | -0.114 | 0 | NO | Cytoplasmic |
| ObPKS2.7 | 4 | 9929 | 1083712.38 | 5.04 | 42.27 | 90.57 | -0.108 | 0 | NO | Cytoplasmic |
| ObPKS2.3 | 4 | 10325 | 1126156.68 | 5.07 | 40.46 | 91.04 | -0.098 | 0 | NO | Cytoplasmic |
| ObPKS1.1 | 1 | 11936 | 1305464.74 | 5.14 | 41.29 | 91.75 | -0.076 | 6 | NO | PlasmaMembrane |
| ObPKS6 | 45 | 14309 | 1566407.09 | 5.21 | 41.12 | 92.03 | -0.075 | 6 | NO | Cytoplasmic |
